# NLRP3 Inflammasome Involvement in Heart, Liver, and Lung Diseases—A Lesson from Cytokine Storm Syndrome

**DOI:** 10.3390/ijms242316556

**Published:** 2023-11-21

**Authors:** Cecilia Napodano, Valeria Carnazzo, Valerio Basile, Krizia Pocino, Annunziata Stefanile, Stefania Gallucci, Patrizia Natali, Umberto Basile, Mariapaola Marino

**Affiliations:** 1 Department of Laboratory of Medicine and Pathology, S. Agostino Estense Hospital, 41126 Modena, Italy; cecilia.napodano@gmail.com; 2 Department of Clinical Pathology, Santa Maria Goretti Hospital, AUSL Latina, 04100 Latina, Italy; v.carnazzo@ausl.latina.it (V.C.); u.basile@ausl.latina.it (U.B.); 3 Clinical Pathology Unit and Cancer Biobank, Department of Research and Advanced Technologies, IRCCS Regina Elena National Cancer Institute, 00144 Rome, Italy; valeriobasile90@gmail.com; 4 Unità Operativa Complessa di Patologia Clinica, Ospedale Generale di Zona San Pietro Fatebenefratelli, 00189 Rome, Italy; krizia.pocino@gmail.com (K.P.); stefanile.nunzia@gmail.com (A.S.); 5 Laboratory of Dendritic Cell Biology, Division of Innate Immunity, Department of Medicine, UMass Chan Medical School, Worcester, MA 01655, USA; stefania.gallucci@umassmed.edu; 6 Diagnostic Hematology and Clinical Genomics, Department of Laboratory Medicine and Pathology, AUSL/AOU Modena, 41124 Modena, Italy; p.natali@ausl.mo.it; 7 Dipartimento di Medicina e Chirurgia Traslazionale, Sezione di Patologia Generale, Fondazione Policlinico Universitario “A. Gemelli” IRCCS, Università Cattolica del Sacro Cuore, 00168 Rome, Italy

**Keywords:** NLRP3 inflammasome, PAMP, DAMP, caspase-1, cytokines, IL-1 β, IL-18, IL-6, pyroptosis, heart, liver, lung

## Abstract

Inflammation and inflammasomes have been proposed as important regulators of the host–microorganism interaction, playing a key role in morbidity and mortality due to the coronavirus disease 2019 (COVID-19) in subjects with chronic conditions and compromised immune system. The inflammasome consists of a multiprotein complex that finely regulates the activation of caspase-1 and the production and secretion of potent pro-inflammatory cytokines such as IL-1β and IL-18. The pyrin containing NOD (nucleotide-binding oligomerization domain) like receptor (NLRP) is a family of intracellular receptors, sensing patterns associated to pathogens or danger signals and NLRP3 inflammasome is the most deeply analyzed for its involvement in the innate and adaptive immune system as well as its contribution to several autoinflammatory and autoimmune diseases. It is highly expressed in leukocytes and up-regulated in sentinel cells upon inflammatory stimuli. NLRP3 expression has also been reported in B and T lymphocytes, in epithelial cells of oral and genital mucosa, in specific parenchymal cells as cardiomyocytes, and keratinocytes, and chondrocytes. It is well known that a dysregulated activation of the inflammasome is involved in the pathogenesis of different disorders that share the common red line of inflammation in their pathogenetic fingerprint. Here, we review the potential roles of the NLRP3 inflammasome in cardiovascular events, liver damage, pulmonary diseases, and in that wide range of systemic inflammatory syndromes named as a cytokine storm.

## 1. Receptors of Innate Immune Response

The main role of the human immune system is to maintain homeostatic tissue function by responding to harmful stress stimuli and infectious agents. It is well known that stereotypical pathogen- and damage-associated molecular patterns (PAMPs and DAMPs) immediately activate innate immunity, evolutionarily older, while the adaptative immune response, more recently evolved, requires specific pathways of interactions and, therefore, more time for activation. Detection of specific PAMPs and DAMPs by host pattern recognition receptors (PRRs) triggers downstream signaling pathways that collectively work to limit pathogens replication as well as cellular damage and modulate innate and adaptive immune responses [1,2]. The PRRs are expressed by immune-competent “sentinel” cells as dendritic cells, macrophages, and mast cells that, in peripheral tissues, are devoted to sense signals referring to pathogens or damage. At least four different PRR families are engaged to recognize harmful signals, such as the toll-like receptor (TLR) family in the plasma and endosomal membranes, where they survey the extracellular space for PAMPs and DAMPs [3]. Therefore, membrane-related PRRs that include TLRs and C-type lectin receptors (CLRs) are devoted to sense PAMPs in the extracellular environment and endosomal compartments, while cytosol is controlled by PRRs such as the NOD (nucleotide-binding oligomerization domain)-like receptors (NLRs), the retinoic acid inducible gene I (RIG-I)-like receptor (RLR), as well as the hemopoietic IFN-inducible nuclear protein with the 200-amino-acid repeat (HIN-200) families [4,5,6]. Most of these receptors induce activation of transcriptional pathways for inflammatory molecules; in addition, members of NLR family, in response to pathogen and danger signals, trigger inflammasomes [7].

The Inflammasomes, cytosolic multiprotein complexes, play the role of for the maturation and activation of the inflammatory caspase-1. While the cascade of events activated by canonical inflammasome culminates with the cleavage of caspase-1, the non-canonical inflammasomes induce activation of caspase 4 and 5 in humans and caspase-11 in mice [8]. Inflammasome activation involves the recruitment of pre-existing caspase zymogens in the protein complex and their auto-activation, which triggers the cleavage and release of interleukins provoking the activation of critical signaling cascades inducing pyroptosis, a proinflammatory cell death mode [8]. The NLR is a family of innate immune receptors located in the cytoplasm of immune cells [9] as inactive monomers. The C-terminal leucine-rich repeat (LRR) domain is the sensor for ligand detection; the central nucleotide-binding and oligomerization domain, also known as NOD or NACHT (which stands for neuronal apoptosis inhibitor protein -NAIP-, MHC class II transcription activator -CIITA-, incompatibility locus protein from Podospora anserine -HET E- and telomerase-associated protein -TP1-), together with NACHT-associated domain (NAD), is involved in ATP-dependent self-oligomerization. Based on the N-terminal domain, which is variable and mediates homotypic interactions with other proteins, the NLR receptor family is divided into five groups [8]. The NLRA group is characterized by an acidic transactivation domain; NLRB displays baculovirus inhibitor repeats (BIRs); NLRC is specifically characterized by the presence of a caspase recruitment domain (CARD); NLRP, which is the largest group, is characterized by the presence of a pyrin domain (PYD); the NLRX group, characterized by atypical N-terminal domain with a mitochondrial-targeting sequence, contains NLRX1, a protein that lacks both PYD and CARD domains [10,11]. N-terminal domains like PYD or CARD, belonging to the six-helix death domain-fold superfamily, are crucial for mediating signaling pathways and triggering inflammation and cell death through the activation of specific caspases [8].

## 2. Mechanisms of Inflammasome Activation and Regulation

**Figure 1:**
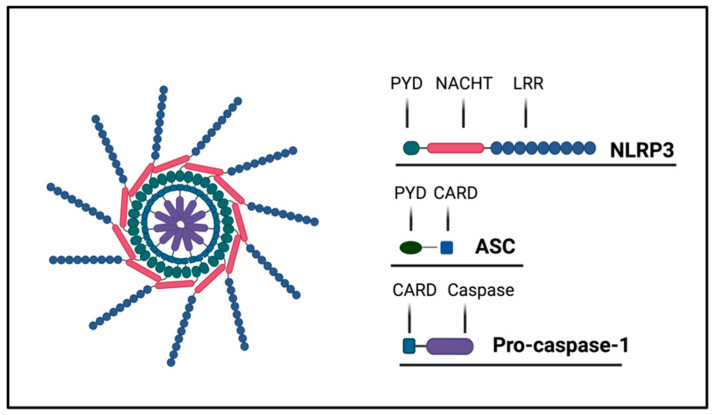
Inflammasome structure. The NLRP3 inflammasome is a complex formed following the interactions among NLRP3, ASC, and pro-caspase 1. NLRP3 is composed by three domains: the pyrin domain (PYD), the NACHT domain, and the leucine-rich repeat domain (LRR). NLRP3 recruits ASCs through PYD–PYD interactions. ASC recruits pro-caspase-1 by CARD–CARD interactions.

The best known NLR is NLRP3, and many characteristics of the family of NLRs have been discovered with this PRR. Since different NLRs have different N-terminal domains (i.e., CARD or pyrin), the molecular steps to induce the activation of caspase-1 can be different. In physiological conditions, NLRP3 is inactive in the cytosol because the LRR is blocked by chaperone proteins. After binding a ligand, NLRP3 is activated and starts the process of oligomerization that culminates in the polymerization of these receptors and binding via pyrin domains to the adaptor protein apoptosis-associated speck-like protein-containing CARD (ASC). ASC, which in humans is encoded by the *PYCARD* gene, is an adaptor molecule located in the insoluble cytosolic fraction [12]. Using its CARD domain, ASC binds monomers of pro-caspase-1 (Figure 1). Therefore, through its N-terminal PYD domain, ASC interacts directly with different PRRs, such as NLRPs, NLRC, or absent in melanoma-2 (AIM2), to assemble the platforms for activation of caspase 1named inflammasomes [13,14]. AIM2 is a cytoplasmic sensor showing an N-terminal PYD domain that, in presence of a double stranded DNA in the cytosol, may form an inflammasome [15]. Other non-NLR proteins mediate this effect: the interferon-inducible protein 16 (IFI-16) [16], the RIG-1 [17], and pyrin [8]. The active inflammasome consists in an oligomer of NLRs forming a structure to a ring that initiates caspase-1 self-cleavage and forms the active tetrameric caspase-1.

**Figure 2:**
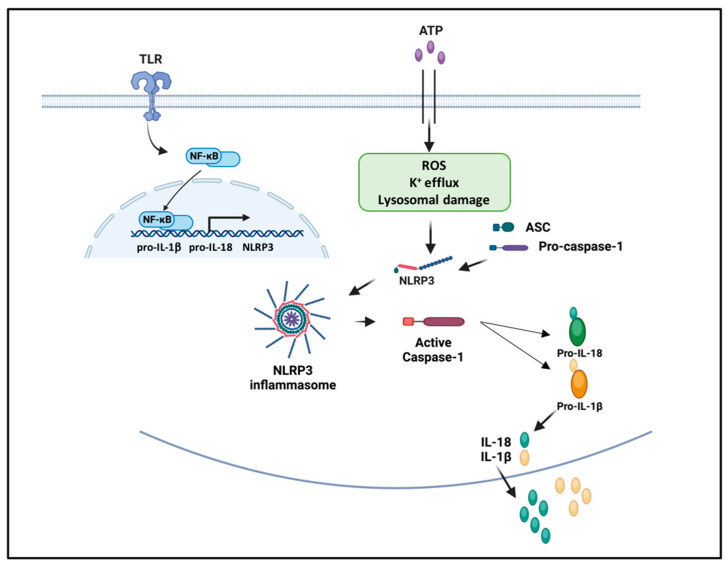
Mechanisms of inflammasome activation and regulation. The priming step is induced by the binding of TLRs to microbial components or by endogenous cytokines like TNF and leads to the transcription of NLRP3 and pro-IL-1β and pro-IL-18 through the activation of the transcription factor NF-kB. The trigger step is induced by several PAMPs and DAMPs and leads to the oligomerization of NLRP3 and the assembly of the ASC-caspase-1 inflammasome, which cleaves pro-IL-1β and pro-IL-18 into active IL-1β and IL-18. Signals of activation are provided by a plethora of stimuli, such as ATP, ROS, viral RNA, and lysosomal damage.

Activation of the inflammasome is a crucial component of the innate immune response, essential to sense different PAMPs and DAMPs generated during infective and non-infective damage. However, excessive activation of the inflammasome may become an important player in the pathogenesis of autoimmune, autoinflammatory, and metabolic disorders. A wide range of stimuli, which are structurally and chemically dissimilar, activate the inflammasome. The NLRP3 inflammasome is formed in response to different DAMPs and PAMPs and to a wide range of trigger signals from the cells’ surface, requiring two important steps. The priming step is induced by the binding of TLRs to microbial components, such as LPS or the nucleic acids of pathogens, activating inflammation though the transcription of nuclear factor NF-kB that promotes the expression of proinflammatory cytokines pro-IL-1β and pro-IL-18 and the transcription of NLRP3 [18]. The trigger step is induced by several PAMPs and DAMPs and leads to the oligomerization of NLRP3 and the assembly of ASC-caspase-1 inflammasome, which cleaves pro-IL-1β and pro-IL-18 into active IL-1β and IL-18. Currently, it is well defined that NLRP3 activation is finely regulated by multiple post-translational modifications and interacting molecules [19] and activated by a wide range of stimuli, including ionic flux (as efflux of potassium), extracellular ATP, mitochondrial dysfunction, production of reactive oxygen species (ROS), lysosomal damage with the release of proteases like cathepsin-B, cholesterol crystals [19], and uric acid [2] (Figure 2).

The efflux of potassium is a requirement for the formation of the NRLP3 inflammasome, and it can be stimulated by toxins or ATP molecules that active the potassium channels causing potassium to leave the cells. Moreover, many microbial stimuli can also trigger the assembly of the inflammasome, including molecules from gram-positive and gram-negative bacteria, viruses, and fungi [20].

In addition to the production of inflammatory cytokines, activation of the NLRP3 inflammasome leads to pyroptosis, which is a rapid and pro-inflammatory programmed cell death. Tight control of inflammasome activity is, therefore, essential and occurs at multiple levels.

## 3. Pyroptosis: The Death Side of Inflammasomes

**Figure 3:**
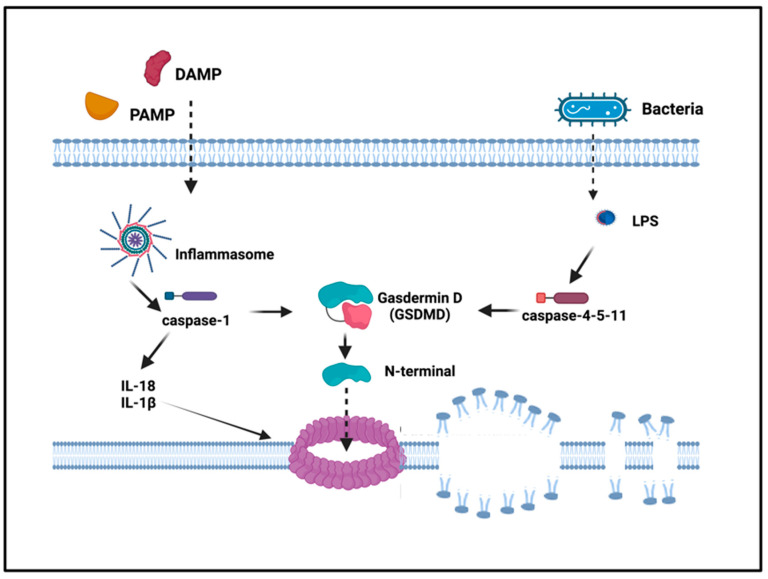
Pyroptosis. In the canonical pyroptosis pathway, intracellular PRRs sense the signals of the pathogens and bind to pro-caspase-1 through the adaptor ASC, forming an inflammasome complex that activates caspase-1. In the non-canonical pathway, intracellular LPS directly binds and activates caspase-4-5-11 to initiate pyroptosis. After caspase activation, pro-IL1β and pro-IL-18 are cleaved and activated to IL-1β and IL-18. Activated caspases cleave gasdermin D, generating an N-terminal cleavage product (GSDMD-NT) that form pores into the membrane, triggering inflammatory death (pyroptosis).

Unlike apoptosis, pyroptosis is a more recently identified form of necrotic and inflammatory programmed cell death induced by inflammatory caspases that occurs most frequently upon microbial infection (intracellular pathogens) and danger signals. In fact, pyroptosis has been defined as a critical mechanism by which inflammasomes contribute to host responses against viruses and gram-negative bacterial pathogens, preventing intracellular replication of infectious agents via the elimination of the infected macrophages and dendritic cells altogether [21,22]. Initially proposed to ascribe the inflammatory nature to caspase-1-dependent cell death, the term of pyroptosis has now been broadened to include cell death driven by non-canonical inflammatory caspases, (caspases 1, 4, 5 in humans and 11 in mice). Recent studies have demonstrated that gasdermin D (GSDMD) mediates pyroptosis following the activation of the inflammasome [23,24]. GSDMD contains an N-terminal cell death domain (GSDMD-NT), a central short linker region, and a C-terminal autoinhibition domain. Activated caspases cleave GSDMD, generating an N-terminal cleavage product (GSDMD-NT) that oligomerizes and inserts into the plasma membrane forming a pore, thus triggering pyroptosis, the inflammatory type of cell death [25] (Figure 3).

In this way, GSDMD-dependent pyroptosis, which facilitates the release IL-1β and IL-18 via unconventional secretion, increases the release of inflammatory mediators, worsening inflammation.

Differently from apoptosis that is not associated with cell lysis, during pyroptosis, water influx inside the cells leads to swelling and to plasma membrane rupture [26]. The subsequent release of cytoplasmic content into the extracellular space acts as a proinflammatory stimulus, differently from apoptosis that is considered immunologically silent [27]. Unlike apoptosis, pyroptosis is restricted to professional phagocytes, macrophages, dendritic cells, and neutrophils, but it has been also described in different cell types as expressing higher levels of the inflammatory caspases such as CD4+ T-cells and keratinocytes. It requires specific biochemical characteristics such as the equipment for caspase activity (although the caspases involved are different), nuclear compartment condensation and oligonucleosomal fragmentation of genomic DNA [22,26].

Furthermore, GSDMD-NT shows bactericidal activity in vitro by binding to cardiolipin residues both in the external and in the internal bacterial membranes. Cardiolipin is also present in internal and external mitochondrial membranes, but it is unclear if, after NLRP3 activation, GSDMD-NT can permeabilize mitochondria membranes by binding to cardiolipin [28].

## 4. Inflammasome Involvement in Cardiovascular Diseases

It is well known that any inflammatory event in cardiac parenchyma may result in fatal consequences for patients (arrhythmia, sudden death, or heart failure) due to the limited capability of regenerating cardiac cells [29].

The most widely characterized inflammasome sensor in the heart is NLRP3, which is activated in response to noninfectious stimuli, such as cell debris during acute myocardial infarction, activating an inflammation that is defined as sterile [30]. Necrotic cell debris induce the inflammasome to assemble and trigger the signaling cascade that culminates with the cleavage of pro-caspase 1 and activation. Pro-inflammatory cytokines promote leukocyte–endothelial cell adhesions, and chemokines drive leukocytes to the infarcted area; TGF-β and IL-10 can hamper inflammation, reducing IL-1β-induced adhesion molecules, promoting cardiac repair by enhancing fibroblast-to-myofibroblast transition, and stimulating extracellular matrix deposition [31,32]. From this point, the persistence of stimulus in tissue drives the outcome of inflammation. The monocyte chemotactic protein 1 (MCP-1) is increased in post-myocardial infarction areas [33]; this chemoattractant gradient for monocytes/macrophages could be sustained through a circuit involving IL-6 that, secreted by damaged cells, binds the soluble circulating receptor IL-6R forming a complex that trigger signals through the membrane gp130, making injured cells responsive to IL-6 (transsignaling of IL-6), and inducing them to produce MCP-1 [34].

**Figure 4:**
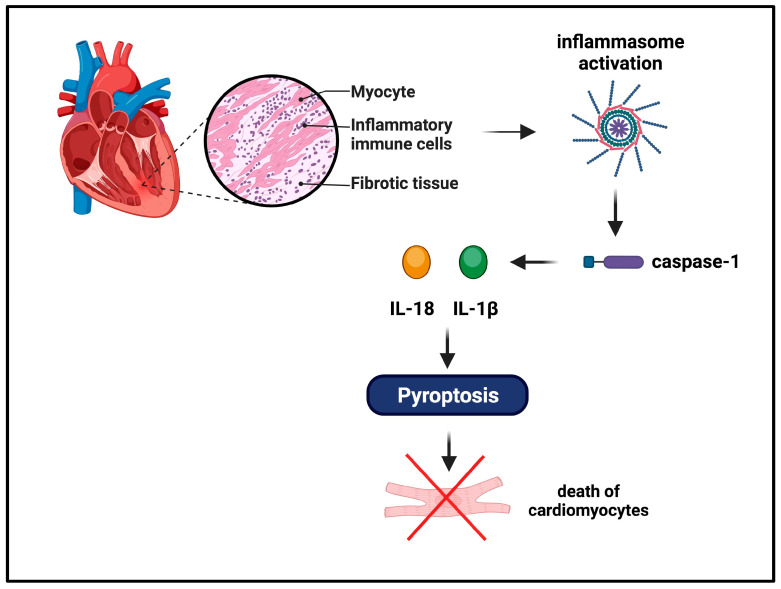
Inflammasome involvement in cardiovascular diseases. Necrotic cell debris induces the inflammasome to assemble and trigger the signaling cascade that activates caspase 1. Inflammatory leukocytes are chemoattracted to the infarcted area; TGF-β and IL-10 can hamper inflammation and promote cardiac repair by enhancing fibroblast proliferation and collagen deposition. Inflammasome activation leads to pyroptosis and death of cardiomyocytes.

Inflammasome activation leads to pyroptosis, the highly regulated inflammatory cell death described above, which represents a harmful stimulus for enhancing inflammation [35]. IL-1β is the prototypical proinflammatory cytokine within the inflammasome that induces cell death in cardiomyocytes (Figure 4). As consequence, strategies aimed to inhibit IL-1 reduce pyroptosis of cardiomyocytes provoked by ischemia, limiting the worsening progression towards cardiac dysfunction [36]. While myocardial injury is always associated with inflammation, the strength of this response vary according to individual features and may be assessed as marker of a negative prognosis [37].

Fibroblasts and myocytes resident in cardiac tissue show high levels of NLRP3 inflammasome, suggesting that the complex is highly involved in ischemic and non-ischemic inflammatory-mediated cardiac damage [38]. In a specific model of calcineurin transgenic mice (CN-Tg) experiencing signs of cardiac injury, the impairment of function (from hypertrophy to dysfunctional dilatation up to cell death) is paralleled by increased expression of NLRP3 in cardiac cells and circulating high levels of IL-1β, the two biomarkers indicating activation of inflammasome priming and triggering steps. In this setting, the employment of IL-1 receptor antagonist (IL-1Ra), acting as competitor for the induction of inflammatory exudate, can counteract the progression towards cell death and heart failure in CN-Tg mice [39].

The activation of the NLRP3 inflammasome also plays a pathogenetic role in atrial fibrillation: in affected patients, IL-1, IL-18, and TNF-α levels positively correlate with the progression of disease [40,41]; while NLRP3, ASC, and pro-caspase 1 levels remain unchanged, caspase 1-p20 protein levels are increased. In patients with atrial fibrillation, the NLRP3 inflammasome is up-regulated, and its activity in the cardiomyocyte correlates with the progression of atrial fibrillation to more persistent forms [42]. To demonstrate the direct involvement and prominent role of inflammatory signaling in atrial fibrillation promotion, Yao et al. set up a model of cardiomyocyte-specific knock-in mice that developed spontaneous premature atrial contractions and inducible atrial fibrillation, attenuated by the administration of a specific NLRP3-inflammasome inhibitor [42].

It has been demonstrated that ionizing radiation could cause cardiovascular injury, and NLRP3 inflammasome up-regulation and activation seem to play a pathogenetic role in radiation damage [43]. Cancer survivor patients, treated with conventional fractionated radiotherapy during childhood, are at greater risk of cardiac death than the general population [44]. Patients with Hodgkin’s lymphoma treated with radiotherapy showed a significant increase in myocardial infarction, angina, valve disease, and congestive heart failure [45]. Fibrosis of myocardium and atherosclerosis represent the two typical manifestations occurring after radiation. Coronary arteries are almost completely narrowed, and tricuspid valve thickening occurs in over 80% of patients who experience irradiation. Because of radiotherapy, patients display pericardial and epicardial fibrosis for the great majority, and pericardial tamponade may occur [46,47,48].

NLRP3 inflammasomes may be involved through different pathways: radiation-related potassium ion efflux and calcium flux [49,50]; up-regulation of cellular ceramide that acts as a second messenger in triggering intrinsic apoptosis [51]; radiolysis of water that generates ROS as well as mitochondrial dysfunction with a consequent release of ROS in the cytoplasm; ultimately, irradiation damage generates DAMPs, resulting in activation of NLP3 inflammasomes and pyroptosis [52].

Endothelium dysfunction allowing LDL-cholesterol accumulation, foam cell formation, and leukocyte infiltration characterizes the pathogenesis of atherosclerosis [53]. Crystals of cholesterol, putative inducers of lysosomal damage, are sensed as DAMPs by NLRP3 expressed in macrophages. In addition, oxidized LDL activates transcription of NLRP3 and pro-IL-1β, promoting atherosclerosis [54]. Accordingly, many evidences strengthen the pathogenetic role of inflammasome and that its blockade rescue from atherosclerosis: increased levels of mRNA for NLRP3, ASC, caspase-1, IL-1β, and IL-18 in plaques [55]; high levels of IL-1β and IL-1 receptor 1 (IL-1R1) within atherosclerotic arteries [56]; on the other side, raised levels of IL-1Ra so as lack of expression of IL-18 in association with reduction of atherosclerosis in coronaries and in a murine model of atherosclerosis respectively [56,57,58].

Risk factors for cardiovascular diseases are age-dependent, ranging from hypertension, atherosclerosis, and heart failure, including acute complications. ‘Inflamm-aging’ is the term coined to depict the link between inflammation and age-dependent inflammatory diseases that describes a low-grade chronic and sterile inflammatory state assessed in elderly though the detection of high level of circulating pro-inflammatory mediators, such as interleukin IL-1β, IL-6, TNF-α, and C-reactive protein (CRP) [59,60,61], useful biomarkers of innate immune system chronic activation [60,61]. Inflamm-aging represents an independent risk factor for cardiovascular diseases, in addition to other co-existing age-dependent impairing conditions such as diabetes, obesity, hypertension, that concur to aggravate their harmful consequences. Obesity and insulin resistance create the pro-inflammatory circuit that promotes diabetes, a well-defined age-dependent cardiovascular risk factor. The increased amount of ROS, together with the cytokines burden, glucose and oxidized lipids favor endothelial dysfunction, with worsening progression towards cardiovascular impairment [62].

Marín-Aguilar et al. showed that ablation of the NLRP3 inflammasome in mice reduced telomere shortening and rescued from the setting of age-related insulin resistance, with a reduction of leptin/adiponectin ratio, IGF-1 levels, and decreased cardiac damage; also the prolongation of PR interval in ECG, marker of age-associated atrial fibrillation, was prevent [63].

Inhibitors that selectively target the formation of the NLRP3 inflammasome, preventing the cleavage of pro-caspase 1, and the downstream effectors (activation of IL-1β) are widely desired and currently in development for the treatment of cardiovascular diseases [64]. At present, three biological inhibitors of IL-1 have been approved for treatment of rheumatoid arthritis and autoinflammatory diseases with promising results for cardiovascular disorders as well: anakinra, a recombinant molecule of the natural inhibitor IL-1 receptor antagonist; canakinumab, a humanized monoclonal antibody neutralizing human IL-1β; and rilonacept, a soluble chimeric Fc fusion protein of IL-1R1 and IL-1R3, able to inhibit the response to both IL-1α and IL-1β [65]. In the large phase III clinical CANTOS trial (Canakinumab Anti-Inflammatory Thrombosis Outcomes Study) including 10 061 patients worldwide, strategies aimed to block IL-1β action decreased the recurrence of ischemia in patients already affected by acute myocardial infarction, while in phase II clinical VCUART trials (Virginia Commonwealth University Anakinra Remodeling Trials), anakinra was significantly associated with lower incidence of new cases of heart failure (HF) and of HF hospitalization. Moreover, anakinra provided positive results in patients with recurrent/refractory pericarditis, as did rilonacept [65]. All these evidences indicate inflammasome or its ultimate effectors as IL-1β as valuable targets to limit adverse effect of ischemic-induced inflammation in myocardium.

## 5. Inflammasomes and Liver Disease

The liver can be exposed to infective pathogens, and, during these events, the inflammasomes play a central role in eliciting adaptive immunity [66]. It has been demonstrated that the NLRC4 is the sensor of bacterial infections in the liver, promoting the release of IL-18, which triggers NK-mediated cytotoxicity [67]. Hepatocytes are targeted by different viruses that lead to the pathogenesis of virus-related liver diseases. Viral infections trigger innate immune responses, which limit viral spread and elicit mechanisms of adaptive immunity for complete removal. Type I interferons together with IL-1β and IL-18 are the key players. Type I interferons promote an antiviral state, and pro-inflammatory cytokines amplify inflammation. The activation of the NLRP3 inflammasome during hepatitis C virus (HCV) infection can have different functions according to the involved cell type, macrophages, hepatocytes, and monocytes [66,68]. Kupffer cells have been identified as the primary cell source of IL-1β in the course of HCV infection, which is essential for amplifying inflammatory responses. Chattergoon et al. demonstrated that the activation of the inflammasome pathway in human monocytes and macrophages during HCV infection is dependent on clathrin-mediated endocytosis; this allows detection of viruses regardless of their ability to infect these cell types [68]. NLRP3 triggers the release of active IL-18, which stimulates the production of IFN-γ in monocytes mediating resistance to HCV. At the same time, the NLRP3 inflammasome stimulates the formation of lipid droplets in hepatocytes, promoting HCV replication and contributing to the pathogenesis of liver diseases [66].

Patients with an active and untreated chronic hepatitis B virus (HBV) infection display higher levels of NLRP3, caspase-1, and IL-1β in comparison to the ones in chronic remission. IL-1β plays a central role during HBV hepatitis onset and in determining the severity of liver inflammation [66,69].

The inflammasome is also involved in the defense of hepatocytes against oxidative stress-induced injury. Following ROS-mediated liver injury, nuclear and mitochondrial DNA (mtDNA) released into the cytosol act as DAMPs to activate innate immunity [70]. Sun et al. demonstrated that caspase 1 is a central driver of mitochondrial autophagy in response to mtDNA depicting a protective mechanism against hypoxia/reoxygenation damage in liver [71]. Moreover, they stressed the role of the AIM2 inflammasome acting as a sensor of innate immunity, leading to autophagy, a hepatoprotective response against oxidative stress-induced injury [72].

**Figure 5:**
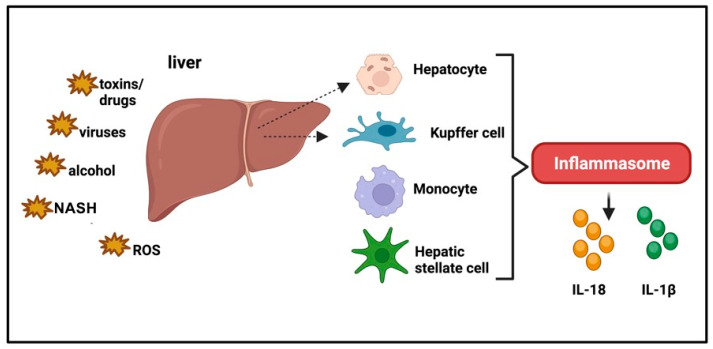
Inflammasomes and liver disease. Inflammasomes are triggered by hepatotropic infective agents but are also involved in the pathogenesis of acute liver injury induced by toxins, drugs, or ischemia/reperfusion, or in chronic liver diseases including alcoholic and non-alcoholic steatohepatitis (NASH).

NLRP3 activation is also involved in initiating and promoting the development of nonalcoholic steatohepatitis (NASH) [73]; in vivo and in vitro experimental studies demonstrated that activation of the NLRP3 inflammasome is mainly associated with NASH, but not with steatosis [74] (Figure 5). Accordingly, it has been reported that gene expression of NLRP3 inflammasome components, pro-IL-18 and pro-IL-1β, is markedly increased in the liver of NASH patients [75,76]. Understanding the implication of inflammasomes in NASH may pave the way for improving a targeted and adequate therapeutic strategy.

## 6. Inflammasome Involvement in Pulmonary Diseases

The inflammasome plays a crucial role in the pathogenesis of acute and chronic respiratory diseases [77]. As in other organ-specific disorders, the NLRP3 inflammasome is first activated by microbial agents, then followed by a second signal that can be triggered by a wide range of factors acting as DAMPs such as extracellular ATP, toxins, RNA viruses, ion flow, mitochondrial dysfunction, ROS, or lysosomal damage, all occurring during pulmonary inflammation.

**Figure 6:**
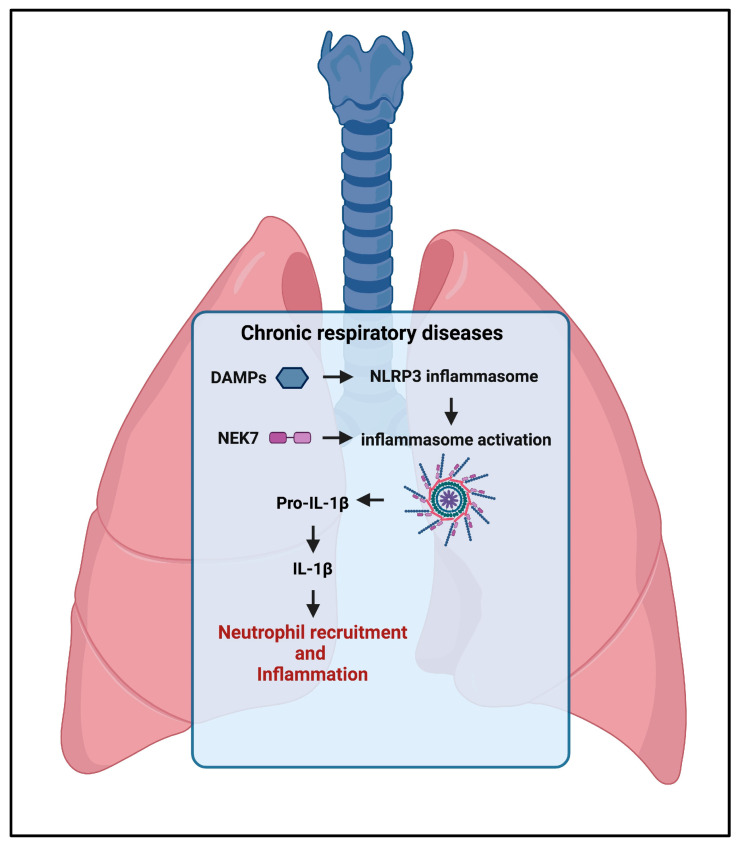
Inflammasome involvement in pulmonary diseases. NEK7 intervenes in the activation of the NLRP3 inflammasome involved in ventilation-induced lung injury. Activation of the NLRP3 inflammasome drives the production of IL-1β and IL-18, which locally provokes an inflammatory reaction with infiltration of immune cells leading to chronic lung injury and pulmonary fibrosis.

A recent study has shown that NEK7 (NIMA-related kinase 7) intervenes in the activation of the NLRP3 inflammasome involved in ventilation-induced lung injury (VILI); mechanical ventilation, a lifesaving treatment for patients who experience respiratory failure, may be complicated by infiltrations of inflammatory cells due to an increase in permeability in the capillary membrane and, consequently, pulmonary edema (Figure 6) [78]. NEK7 is a highly conserved multifunctional protein kinase belonging to the NEK family, expressed in eukaryotic cells, mainly regulating the G2 phase and the mitosis of the cell cycle. Upon sensing induced NLRP3 conformational change, NLRP3 directly binds NEK7, and this interaction enables NLRP3 inflammasome activation, inducing formation of an NLRP3 inflammasome disk and promoting the whole cascade of events culminating in the recruitment and activation of caspase-1 [79].

To better define the role of NEK7 in VILI, the authors reproduced in vitro a mouse model of lung stimulated epithelial cells that were transfected with small interfering RNA for NEK7 and pretreated with oridonin (Ori) or glibenclamide. Ori, a diterpenoid isolated from Rabdosia rubescens, exhibits anti-inflammatory properties in different tissues; glibenclamide, an ATP-sensitive K+ channel inhibitor, is widely used for the treatment of type II diabetes mellitus. The authors’ results demonstrate that while mechanical stress increases NEK7-NLRP3 interaction leading to assembly and activation of NLRP3 inflammasome downstream of potassium efflux, Ori and glibenclamide display an anti-inflammatory function as well as to NEK7 depletion, interfering with potassium efflux and blocking the interaction between NEK7 and NLRP3, improving VILI [78].

D’Amico et al. evaluated the role of the product encoded by the *formyl peptide receptor 1* (*FPR-1*) gene in experimental models of bronchiolitis obliterans syndrome (BOS), a chronic lymphoproliferative lung disease caused by an injury to the small respiratory tract that is considered the main manifestation of chronic lung allograft rejection [80]. *FPR-1* encodes the formyl peptide receptor 1 expressed on phagocytic cells of the immune system [81], mediating their recruitment at the lesion site and activation in the NLRP3 inflammasome.

D’Amico et al. found that specific deletion of *IL-1β*, *IL-18*, *Casp-1*, and *FPR-1* genes reduce tissue damage and lung inflammation. In *FPR-1* KO mice, they found a reduction in histological markers of BOS, poor resistance to cell death, and a reduction in the number of immune cells and in nitrotyrosine, PARP, VEGF, and TGF-β markers compared to wild-type mice with a protective effect against BOS damage. Furthermore, in the absence of the *FPR-1* gene, the nuclear translocation of NF-kB, the activation of the inflammasome NLRP3, and the mitogen-activated protein kinase pathway (MAPK) are significantly reduced [80].

Kasper and Barth demonstrated the substantial contribution of the purinergic receptor (P2X7R) to the pathogenesis of pulmonary fibrosis, its central role in activating NLPR3, and the turnover of different tight junctions, ultimately, to maintaining the integrity of the alveolar barrier [82]. Damaged alveolar type I cells (AECI) characterize pulmonary fibrosis, incorrectly replaced by hyperplastic proliferation of AECII, which in turn differentiate in type I [82].

The P2X7R expressed in AECI consists of a cationic channel activated by low concentrations of extracellular ATP that allows the flux of mono and divalent cations (Ca^2+^, Na^+^, K^+^) through the plasma membrane; following tissue damage or death, high concentrations of ATP lead to the formation of a large non-selective pore in cells with P2X7R in the membrane. The opening of pores leads to a rapid outflow of K+ ions from the cytosol [83] and triggers the assembly and activation of the inflammasome leading to activation of pro-caspase-1. In a mouse model of lung injury, the P2X7R KO phenotype was more resistant to inflammatory damage [84].

Previous studies have shown that P2X7R and aquaporin 5 (AQP5) take part in the alveolar barrier function. Downregulation of AQP5 and decreased mRNA expression levels of the AQP5 protein with fibrotic areas has been shown in P2X7R knockout animals; therefore, the purinergic receptor has a role in regulating the protein. In addition, isolated murine epithelial cells deficient in AQP5 had increased barrier function activity [85]. Inhibitors that block P2X7R activity have been used in the preclinical phase I and II of clinical studies on patients with inflammatory lung disease [86].

Shao et al. demonstrated that glyburide, an antidiabetic drug, improves ozone-induced lung inflammation and injury by blocking the NLRP3 inflammasome mainly through blocking KATP channels. After 24 h of intratracheally administration of glyburide, they analyzed the bronchoalveolar lavage fluid (BALF) of C57BL/6-treated mice. They found that glyburide inhibited the expression of NLRP3, IL18 and IL-1β, demonstrating the inhibitory effect of glyburide on the NLRP3 inflammasome in lung tissues [87].

Many studies report that the NLRP3 inflammasome is involved in respiratory diseases such as acute lung injury (ALI)—acute respiratory distress syndrome (ARDS) caused by pathogenic microorganisms such as influenza A virus [88], Pseudomonas aeruginosa [89], and Staphylococcus aureus [90].

Recently, it has been shown that erythropoietin (EPO), employed for the treatment of anemia, also demonstrates therapeutic effects in respiratory disorders such as ALI, as it suppresses the NLRP3 inflammasome [91]. In this study, ALI was induced in C57BL/6 mice by intraperitoneal LPS injection, and intraperitoneally administered EPO significantly attenuated LPS-induced lung injury by restoring histopathological changes and protein levels in BALF. EPO exerts its effect through the EPOR/JAK2/STAT3/NF-kB pathway in mice; EPO binds to its receptor EPOR, the phosphorylation of JAK2 (p-JAK2) activates and phosphorylates STAT3 (p-STAT3), which in turn inhibits the phosphorylation of NF-kB p65 and, consequently, its translocation into the nucleus and the transcriptional activity of NF-kB p-p65. In this way, the transcription and expression of NLRP3 and pro-IL-1β are inhibited, and the concentration of IL-1β is reduced.

## 7. The Inflammasome in the Pathogenesis of Cytokine Storm Syndrome

**Figure 7:**
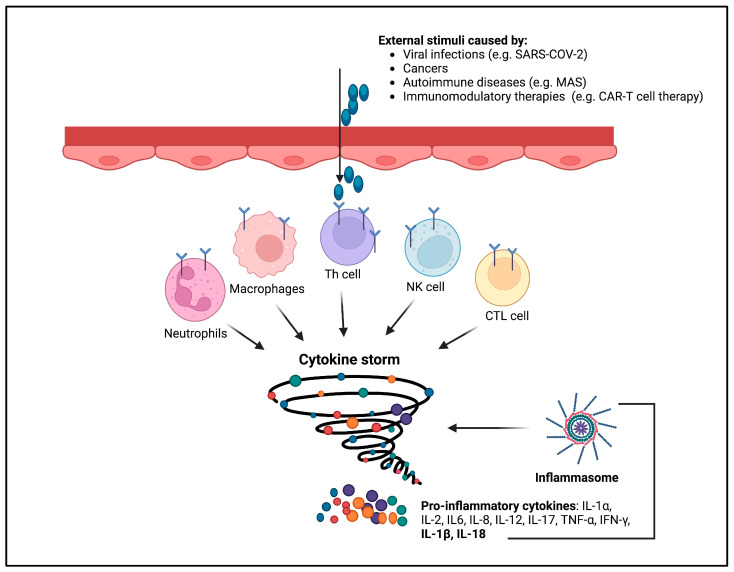
Cytokine storm. In several diseases such as viral infections, cancers, autoimmune diseases, and immunomodulatory therapies, elevated levels of proinflammatory cytokines have been detected. This process is referred to as a cytokine storm, a consequence of the overactivation of the NLRP3 inflammasome and results in excessive cytokine production.

The activation of the inflammasome has also been involved in the pathogenesis of life-threatening systemic inflammatory syndromes grouped under the umbrella name of cytokine storm [92]. Although there are no accepted definitions that can capture the entirety of these syndromes, clinical disorders called cytokine storm or cytokine release syndrome are characterized by elevated levels of circulating cytokines, immune hyperactivation, and multi-organ disfunction that can come to be fatal [93]. Blockades of pro-inflammatory cytokines, first IL-6, then interferon-γ and IL-1, have been shown to be therapeutic in many cytokine storms [94,95,96,97,98,99]. A cytokine storm can develop during very different pathological conditions, from viral infections to cancers and autoimmune diseases, and upon immunomodulatory therapies [93]. Although they have different pathogeneses, they share systemic multi-organ damage that is caused by a dysregulated, excessive host immune response rather than by the associated microbial pathogen or endogenous insult [100] (Figure 7).

The clinical presentation of most if not all cytokine storms includes fever and several symptoms of general malaise like fatigue, anorexia, and myalgia [92]. Other signs are more specific of damaged tissues and organs, which can be different in different cytokine storms. Skin rash, arthralgia, hepatomegaly, elevated liver enzymes, and renal dysfunction, or nausea and diarrhea, are often the signs that inflammation is affecting the skin, liver, kidneys, or the gastrointestinal tract. Endothelial damage can be accompanied by increased vascular permeability and coagulopathy that can become disseminated intravascular coagulation [101]. The heart can also be involved, with hypotension, tachycardia up to full cardiomyopathy, and confusion, aphasia, or seizures can be signs of a suffering central nervous system. Finally, pulmonary dysfunction appears as pulmonary edema, dyspnea up to the full ARDS [102].

Classification criteria have been defined for some specific cytokine storms, which share many of the same biomarkers, with differences in levels and associations [103,104]. The presence of pro-inflammatory biomarkers, coagulopathy, and cytopenia with an imbalance in the number of immune cell subpopulations, together with the therapeutic benefit from cytokine blockade and immunosuppression, indicate underlying immunopathologic mechanisms. The interest of the scientific community in investigating these mechanisms has dramatically increased during the ongoing COVID-19 pandemic upon the recognition that the severe cases of SARS-CoV2 infection resemble a new form of cytokine storm [105]. As a testimony of such interest, 9022 publications, between research articles and reviews, mentioned the term cytokine storm between the beginning of 2020 and October 2023, as opposed to the 1709 publications spanning the 31-year period from 1988 to 2019.

The first class of cytokine storm that was classified with this name is an iatrogenic one that develops upon specific immune-modulatory therapies, including CAR T-cell therapy, immunostimulatory monoclonal antibodies, and graft-versus-host disease [102]. It is also called cytokine release syndrome, and it was observed during the massive cell death induced by CAR T-cell treatment of B-cell malignancies. The main culprits were identified as IL-6 and IFN-γ, and IL-6 blockade was shown to resolve most of the pathology [106]. High levels of IL-1β are also found in cytokine release syndrome, and inhibition of IL-1 has been reported to improve the cytokine release syndrome in animal models [96]. The production of these cytokines is considered to derive mostly from macrophages recognizing DAMPs that are released by tumor cells lysed by the CAR T-cells [107].

Indeed, in addition to genetic predisposition, the tumor burden is an important factor associated with the severity of the cytokine storm during CAR T-cell therapy [108]. Other immunotherapies that can trigger a cytokine release storm are T-cell engager antibodies, like the anti-CD19/CD3 antibody blinatumomab [109] and anti-CD28 antibody [110], in which the hyperactivation of T-cells induced by the administered antibodies likely mediates the induction of cytokine release.

Macrophage activation syndrome (MAS) is a cytokine storm associated with autoimmune diseases, like systemic lupus erythematosus, and autoinflammatory conditions like systemic juvenile idiopathic arthritis [111]. An extended definition of MAS also includes cytokine storms associated with infections (herpes viruses), cancer (leukemia), and post-transplantation immunodeficiency [112]. Classification criteria of MAS include the presence of fever, high levels of the pro-inflammatory marker ferritin, and any two of the following laboratory parameters: low platelet count, low fibrinogen, high aspartate transaminase (AST), or high triglycerides, indicative of heightened inflammation, coagulopathy, and liver damage [113]. Similar criteria have been defined for another cytokine storm in which hyperactivated macrophages are central to the disease, i.e., hemophagocytic lymphohistiocytosis (HLH), characterized by persistent pathologic activation of cytotoxic T lymphocytes, NK, and macrophages. HLH can be a genetically driven familial disease, or it can be secondary to malignancies, viral infections, autoimmunity, or yet unknown causes. The HLH classification criteria include fever; splenomegaly; cytopenia affecting ≥ 2 of 3 lineages in peripheral blood (i.e., low hemoglobin, platelets, or neutrophils); hypertriglyceridemia and/or hypofibrinogenemia; hemophagocytosis in bone marrow, spleen or lymph nodes; low NK activity (often difficult to measure outside of research labs); high ferritin; and high soluble CD25 [114]. Such criteria identify a severe process occurring systemically and in major immunological organs, like the spleen and bone marrow, where macrophages are found engorged with mature red blood cells, nucleated erythroblasts, granulocytes, or lymphocytes (hemophagocytosis), as evidence of abnormal innate function and lack of clearance of overactivated innate immune cells [115]. Compared to the criteria of MAS, those of HLH are broader, and, indeed, a recent redefinition of these clinical conditions considers MAS a secondary form of HLH [116].

The genetic component of the cytokine storm, especially MAS or HLH, is becoming better known. Together with mutations associated with defects in cytotoxicity by natural killer and CD8 T-cells [112,117], patients developing familial HLH and recurrent or isolated MAS present mutations causing dysregulated activation of the inflammasome and overproduction of the IL-1 family of cytokines [118,119]. Mutations in the NLRC4 inflammasome were found in patients with recurrent MAS [120], and patients with features of MAS and septic shock had mutations in the inflammasome *NLRP3* and *Mediterranean fever* (*MEFV*) genes, leading to increased production of IL-1β and IL-18 by their monocytes in vitro [121]. Together, these results support an important role for inflammasome gene variants in the risk of MAS and possibly other forms of cytokine storm.

Viral infections are also a well-known trigger of cytokine storms, and severe cases of influenza infection, Epstein-Barr, as well as dengue fever and the hemorrhagic fever Ebola have shown the features of this hyperimmune reaction [122,123,124]. The involvement of the inflammasome in viral infections can be indirect, when it is due to the genetic predisposition described above for MAS and HLH, but it can also be due to the direct hyperactivation of the inflammasome as a pattern recognition receptor by viral proteins and nucleic acids. For example, Influenza A virus proteins NP and PB1 can stimulate NLRP3 inflammasome activation as well as the induction of apoptosis, necroptosis, and pyroptosis via the Z-RNA sensor ZBP1 [125], and protein M of the dengue virus [126] and Ebola infection have also been shown to activate NLRP3 [127].

Severe cases of infection by SARS and SARS-CoV2 can develop a cytokine storm [128]. Many reports have described specific characteristics for the storm occurring during SARS-CoV2 (COVID-19) that share decreased numbers of lymphocytes, high levels of CRP, which is a sign of inflammation and an indirect correlate of high levels of IL-6, and high levels of the lactate dehydrogenase (LDH), sign of cell death [129]. The COVID-19 Research Group of Temple University found that the cytokine storm in COVID-19 patients (COVID-CS) did not fit the classification criteria of MAS or HLH and proposed new predictive criteria [104]. Their investigation showed that, together with fever and signs of malaise and high levels of ferritin and CRP, alterations in laboratory parameters included in three clusters can identify a cytokine storm with a significantly longer length of hospitalization and higher mortality. The first cluster included the decreased levels of albumin and percentage of lymphocytes, and increased absolute numbers of neutrophils, all signs of inflammation; the second cluster included increased levels of the liver enzymes alanine aminotransferase (ALT) and aspartate transaminase (AST), LDH, troponin I, and D-dimers, all signs of tissue damage involving the liver, the cardiovascular system, kidneys, and immune thrombosis. The third cluster included the altered level of electrolytes and blood urea nitrogen (BUN): creatinine ratio and signs of pre-renal electrolyte imbalance. These results highlight the relevance of hyperinflammation, cell death, and tissue damage in the COVID-CS.

Cell death has been implicated in the pathogenesis of many cytokine storms, either as a direct cause of tissue damage, or because it induces the release of DAMPs, which stimulate innate immune cells to secrete pro-inflammatory cytokines [130]. Cell death is also an important cause of endothelial leakage, which can trigger coagulation, amplify inflammation, and possibly induce systemic exposure to bacterial PAMPs, further fueling inflammation and the production of cytokines [131]. Cell death and tissue damage are recognized as major features in the COVID-CS [132], and the recent literature, including the predictive criteria described above [104], highlights that, in addition to markers of inflammation, most of the alterations found in COVID-CS are signs of tissue damage, like high levels of LDH, which is often measured as a proxy for pyroptosis [133]. The role of the inflammasome and pyroptosis in COVID-CS is supported by the increased levels of IL-1β found in the serum of severe patients [134] and the low levels of the inhibitory IL-1Ra associated with higher mortality [135]. Furthermore, some studies have reported the beneficial therapeutic effects on COVID-CS of the IL-1 receptor antagonist anakinra [136,137] or the IL-1 blockade with the biologic canakinumab [138], although these results are balanced by a clinical trial stopped for futility in patients with COVID-19 and mild-to-moderate pneumonia [139,140] and by another clinical trial testing the effects of canakinumab in patients hospitalized for severe COVID-19, in which survival without invasive mechanical ventilation, mortality, and measurements of biomarkers of systemic hyperinflammation were found not statistically different between patients treated with the anti-IL-1β antibody treatment and placebo controls [141]. These conflicting results may be explained by the difference in severity and the small size of some of the evaluated cohorts. Further studies have evaluated the ability of IL-1 blockade to prevent specific tissue damage in patients with COVID-19 infection, such as cardiac injury [142]. At the beginning of September 2021, Kyriazopoulou et al. published beneficial effects of recombinant IL-1Ra anakinra in a phase III randomized controlled trial in moderate to severe COVID-19 pneumonia patients, with decreased mortality and hospital stay [143]. These results were obtained in a cohort that was larger in size compared to the first reports and in which patients were selected using a novel biomarker, soluble urokinase plasminogen activator receptor (suPAR) that was recently found increased in conditions of tissue damage like renal or cardiac injury [144,145] and in severe COVID-19 [146,147]. Since the improvement in mortality was evident in patients who fit the criteria of COVID-CS proposed in [104], which include many signs of cell death, together these results suggest that cell death, and inflammasome-triggered pyroptosis in particular, may play a pathogenic role in fueling the pro-inflammatory response in COVID-CS. It is interesting to speculate that insufficient production of type I interferons found associated with severe COVID-19 infection [148] may play a role in the development of a cytokine storm not only for the insufficient control of viral replication and anti-viral host defense, but also because type I interferons have been shown to limit inflammasome activation [149].

## 8. Conclusions

A circuit involving the NLRP3 inflammasome in cellular players leads to a range of inflammatory disorders. According to this, the characterization of underlining molecular mechanisms leading to NLRP3 overexpression or aberrant activation is widely desired to realize anti-inflammatory precision medicine. This review revealed the recent progress and perspectives of NLRP3 inflammasome involvement associated with organ dysfunction in the heart, liver, and lungs, and its role in systemic inflammatory cytokine reactions characterized by worsening progression and increased mortality. We assessed the current state of the art, considering the therapeutic potential of advanced NLRP3 inflammasome inhibitors.

## Data Availability

Data sharing is not applicable to this article, as no datasets were generated or analyzed during the current study.
